# Awareness and Attitudes Among Parents of Females Aged 9-26 in Saudi Arabia Regarding Human Papillomavirus Vaccination

**DOI:** 10.7759/cureus.62470

**Published:** 2024-06-16

**Authors:** Taif S Alharthi, Renad K Alqahtani, Manar Alghamdi, Abdulaziz A Munshi, Khalid A Alzahrani, Abdulhamid Q Alenezi, Muhjah M Almurakshi, Alanoud Z Aljarbou

**Affiliations:** 1 Department of Medicine, Taif University, Taif, SAU; 2 Department of Medicine, Imam Mohammad Ibn Saud Islamic University, Riyadh, SAU; 3 Department of Medicine, Faculty of Medicine, Umm Al-Qura University, Makkah, SAU; 4 Department of Medicine, Umm Al-Qura University, Makkah, SAU; 5 Department of Emergency, Prince Abdulaziz Bin Musaed Hospital, Arar, SAU; 6 Department of Pediatric Infectious Diseases, Imam Mohammad Ibn Saud Islamic University, Riyadh, SAU

**Keywords:** cervical cancer, vaccine, awareness of human papilloma virus, human papilloma virus vaccination, hpv

## Abstract

Background

Most cases of cervical cancer are caused by the human papillomavirus (HPV) infection, which can be prevented by vaccination. The HPV vaccine received approval in Saudi Arabia in 2010.

Objectives

This study aimed to examine the awareness and attitudes toward the HPV vaccine among parents of females aged 9-26 in Saudi Arabia and explore factors contributing to hesitancy or acceptance.

Methods

Conducted from November 2022 to June 2023 in Saudi Arabia, this cross-sectional study surveyed parents of females aged 9-26 using a self-administered questionnaire. Data collected included parental demographics, maternal vaccination status, awareness of the HPV vaccine, and attitudes toward it.

Results

Out of 551 participants, 445 (82.4%) were mothers. Most participants (331; 69.1%) had attained a university education, and approximately half (315; 57.2%) were employed. A total of 339 (61.5%) were aware of the HPV vaccine, 256 (46.5%) knew of its connection to cervical cancer, and 296 (53.7%) understood its preventive role. Among them, 230 participants demonstrated a good level of awareness, while 321 had a poor level. The majority (377; 68.4%) intended to vaccinate their daughters. Reasons for hesitancy among those unwilling included lack of awareness (234; 42.5%), insufficient information (206; 37.4%), fear of vaccines and needles (203; 36.8%), and conflicting medical opinions (165; 29.9%).

Conclusion

The current level of awareness regarding HPV vaccines within the general population is deemed satisfactory, with the majority expressing intent to vaccinate their daughters. Ongoing efforts are warranted to enhance awareness further, particularly by leveraging social media platforms and the expertise of trusted physicians and healthcare authorities. These endeavors are crucial for mitigating the preventable impact of HPV infection. Additionally, it is imperative to sustain immunization programs for HPV vaccines, ensuring streamlined vaccine administration.

## Introduction

Human papillomavirus (HPV) is the most widespread sexually transmitted infection (STI) globally [1]. According to the WHO, HPV infection ranks as the primary cause of cervical cancer, impacting 300 million women worldwide [2]. Annually, HPV infection is linked to over 311,000 deaths from cervical cancer [3]. In Saudi Arabia, the prevalence of HPV infection among the general population is approximately 9.8%-43% [4]. The WHO's World Health Statistics Report 2010 indicates that the at-risk female population for cervical cancer in Saudi Arabia (aged >15 years) amounts to 6.51 million [5]. Cervical cancer ranks as the eighth most prevalent malignancy among Saudi Arabian women aged 14-44 [6]. Key preventive measures against cervical cancer encompass cytology testing (Pap smears) and HPV vaccinations. Vaccines, such as the bivalent (2vHPV) targeting types 16 and 18 and the quadrivalent (4vHPV) targeting types 6, 11, 16, and 18, demonstrate efficacy against the most prevalent carcinogenic HPV genotypes. Given that 78% of HPV-16 and HPV-18 genotypes account for 92% of cervical tumors in Saudi Arabia, immunization is anticipated to safeguard against over two-thirds of cervical malignancies in this country [6].

As of the end of 2020, 111 countries, primarily high- and middle-income nations, had integrated HPV vaccination into their routine immunization programs. To effectively combat cervical cancer as a global public health concern, ambitious targets for high coverage of HPV vaccination, along with comprehensive screening and treatment of precancerous lesions, must be achieved by 2030 and sustained at this high level for decades if cervical cancer is to be eliminated as a worldwide public health issue [2].

Multiple studies have shown positive parents' knowledge, awareness, and attitude toward HPV vaccination [7,8].

In 2010, Saudi Arabia approved an HPV prophylaxis vaccine for women aged 11-26. Despite this, only a few studies have been conducted in the country to evaluate parents' knowledge, awareness, and attitude toward HPV vaccination, revealing negative attitudes and low levels of awareness [9,10].

Efforts have been made to raise awareness and provide access to the HPV vaccine for young females aged 9-14 in Saudi Arabia. In early April 2022, the Ministry of Health (MOH) initiated a large-scale immunization program [11]. In alignment with this initiative, we aim to assess parental awareness and attitude toward the HPV vaccine following its implementation.

## Materials and methods

Study design and participants

This study aimed to investigate the awareness and attitude toward the HPV vaccine among parents of females aged 9-26 across different regions around Saudi Arabia while also examining the reasons for hesitancy and acceptance among these parents. A cross-sectional study was conducted in the Kingdom of Saudi Arabia from November 2022 to June 2023. It involved parents of females aged 9-26 residing in Saudi Arabia, regardless of whether their daughters had been vaccinated. Exclusions were made for parents of females below the age of nine or above 26 and those who declined participation.

Sample size and recruitment

The Raosoft sample size calculator (Raosoft Inc., Seattle) was employed to calculate the sample size, considering the population size, a 95% confidence level, and a 5% margin of error. The minimum expected sample size was determined to be 550. Data collection was conducted through an online questionnaire-based survey designed using Google Forms. The questionnaire was culturally sensitive and tailored explicitly by the research team and reviewed, modified, and validated by an infectious disease expert.

Data collection

Before data collection, a pilot study involving 20 parents was conducted to refine survey questions and ensure the questionnaire's clarity, consistency, and validity. The questionnaire was structured into four main sections.

The first section encompassed parental demographics, including age and education level. The second section covered the history of maternal vaccination and their daughters' completion of childhood immunization. The third section focused on parental awareness of the HPV vaccine, while the final section explored parental attitudes toward it. The questionnaire was made available in Arabic and distributed electronically through social media.

Data analysis

After collecting the data, we revised, coded, and input the data into Statistical Product and Service Solutions (SPSS, version 22; IBM SPSS Statistics for Windows, Armonk, NY). All statistical analyses were conducted using two-tailed tests, with a significance level set at p < 0.05. To determine overall awareness and perception, scores from individual items were summed. Participants with an overall score of less than 60% of the maximum score were categorized as having poor awareness levels.

In contrast, those scoring 60% or higher were categorized as having a good overall awareness level. Descriptive analysis, including frequency and percentage distributions, was conducted for all variables, encompassing parents' demographic data, education levels, and vaccination histories of both themselves and their daughters. Continuous variables were presented as mean ± standard deviation. Additionally, participants' awareness regarding the HPV vaccine for females aged 9-26 and their hesitancy and acceptance of the vaccine were tabulated alongside their overall awareness level. Simultaneously, sources of information were graphically represented. Cross-tabulation graphs were utilized to examine factors associated with parents' awareness and perception of the HPV vaccine, with statistical testing conducted using Pearson's chi-square test and exact probability test for small frequency distributions.

Ethical considerations

This study received approval from the Al-Imam Muhammad Ibn Saud University Institutional Research Board (IRB) (approval no. 448/2023). A consent form was included at the beginning of the questionnaire to ensure the participants' privacy. Personal identifiers, such as names, national IDs, or any other means of identification, were not collected. Additionally, no incentives or rewards were offered to participants.

## Results

A total of 551 eligible parents participated in the study, with the majority (82.4%; 454) being mothers of the females. The parents' ages ranged from 20 to over 50 years, with a mean age of 42.6 ± 12.7 years. Among the enrolled participants, 95.6% (527) were Saudi nationals, with the largest proportion (40.8%; 225) residing in the Western region.

Over two-thirds of the participants were highly educated, with 381 (69.1%) being university graduates. Additionally, half of the participants (57.2%; 315) reported being employed (Table 1).

**Table 1:** Demographic profile of parents of female children aged 9-26 years in Saudi Arabia (n = 551).

Personal data	No	%
Respondent		
Mother	454	82.4%
Father	97	17.6%
Human papillomavirus		
20–30 years	96	17.4%
31–years 40	177	32.1%
41–years 50	199	36.1%
> Years 50	79	14.3%
Nationality		
Saudi	527	95.6%
Non-Saudi	24	4.4%
Region		
Northern	85	15.4%
Central	90	16.3%
Eastern	82	14.9%
Western	225	40.8%
Southern	69	12.5%
Educational level		
Below secondary	25	4.5%
Secondary	89	16.2%
University	381	69.1%
Postgraduate	56	10.2%
Employment		
Unemployed	226	41.0%
Student	10	1.8%
Employed	315	57.2%

Only 21.8% (118) participants reported that the mother (i.e., the mother of the daughter) had received the HPV vaccine; of those, 81.4% (96) completed the HPV vaccination series. Approximately one-fourth of the participants (26%; 144) stated that their daughters had been previously vaccinated against HPV. Furthermore, 88.4% (487) of the participants' daughters completed their immunizations by the Ministry of Health's recommended schedule (Table 2).

**Table 2:** HPV vaccination history among parents and female children aged 9–26 years in Saudi Arabia (n = 551).

	Count	N %
Has the mother (mother of the daughter) received the HPV vaccine?		
Yes	118	21.4%
No	433	78.6%
Have you completed the HPV vaccination? (n = 118)		
Yes	96	81.4%
No	22	18.6%
Has your daughter received the HPV vaccine previously?		
Yes	144	26.1%
No	407	73.9%
Has your daughter completed her immunizations according to the children's immunization schedule recommended by the Ministry of Health?		
Yes	487	88.4%
No	64	11.6%

**Figure 1:**
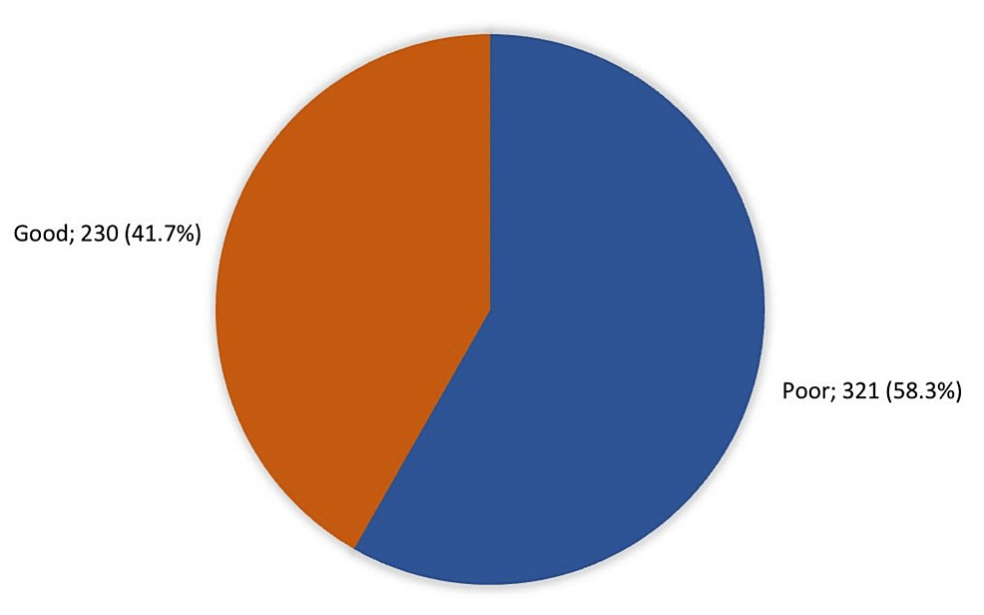
Awareness level of HPV vaccine among parents of females aged 9-26 in Saudi Arabia (n = 551).

Overall, the awareness of HPV was considered satisfactory. Among the participants, 41.7% (230) demonstrated a good overall awareness level regarding the vaccine, while 58.3% (321) exhibited a poor awareness level (Figure 1).

Of the study participants, 61.5% (339) were aware of a vaccine against HPV. Additionally, 46.5% (256) were cognizant of the fact that HPV has been established as the primary cause of cervical cancers, and 53.7% (296) knew that cervical cancer can be prevented with a vaccine. Furthermore, 368 (66.8%) respondents knew the HPV vaccine was recommended for females aged 9-26. Moreover, 235 (42.6%) had heard about the Ministry of Health's campaign (Table 3).

**Table 3:** Parental awareness and attitudes toward HPV vaccination among females aged 9-26 in Saudi Arabia (n = 551). HPV: human papillomavirus; MOH: Ministry of Health

Awareness items	No	%
Are you aware that a vaccine exists for HPV?		
Yes	339	61.5%
No	212	38.5%
Are you aware that HPV has been established as the leading cause of most cervical cancers?		
Yes	256	46.5%
No	295	53.5%
Are you aware that cervical cancer can be prevented through vaccination?		
Yes	296	53.7%
No	255	46.3%
What is the eligible age for the HPV vaccine?		
Birth to nine years	80	14.5%
9–26 years	368	66.8%
26 years or older	103	18.7%
Are you familiar with the MOH's campaign promoting the HPV vaccine for individuals starting at age 9?		
Yes	235	42.6%
No	316	57.4%

**Figure 2:**
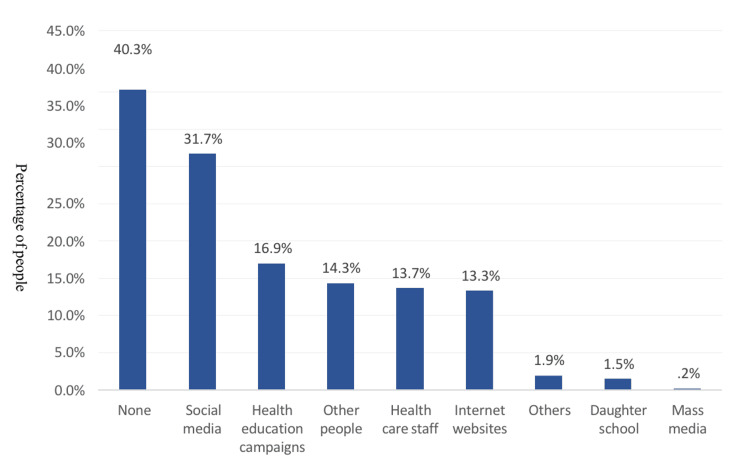
Source of information about human papillomavirus vaccination among study participants (n = 551).

Figure 2 shows the primary sources of information regarding HPV vaccination among the study participants. The main sources cited were social media (31.7%), health education campaigns (16.9%), advice from others (14.3%), healthcare professionals (13.7%), and online resources (13.3%). Notably, 40.3% of participants did not specify a particular source of information.

Over two-thirds of the study's participants, accounting for 68.4% (377) of the parents surveyed, expressed intent to vaccinate their daughters. Among those hesitant, the primary reasons cited included a lack of awareness about the vaccine (42.5%), insufficient information regarding its efficacy (37.4%), fear of vaccines and needles (36.8%), conflicting advice from healthcare providers (29.9%), and lack of recommendation from their physician (9.2%). Conversely, among parents inclined to accept vaccination, the leading reasons included endorsement by a physician (61.8%), confidence in the vaccine's effectiveness (48.8%), and assurance of its safety (47.5%), as outlined in Table 4.

**Table 4:** Parental hesitancy and acceptance regarding the HPV vaccine for females aged 9–26 (n = 551). HPV: human papillomavirus; MOH: Ministry of Health

Hesitancy regarding HPV vaccination	No	%
If your daughter has not received the HPV vaccine yet, do you intend to have her vaccinated?		
Yes	377	68.4%
No	174	31.6%
What are the factors contributing to hesitancy in administering the HPV vaccine to daughters?		
I have never heard of the vaccine before	74	42.5%
Lack of information about the vaccine	65	37.4%
Fear of vaccines and needles	64	36.8%
Experiencing hesitancy owing to conflicting medical advice	52	29.9%
There is no need for my daughter to get vaccinated; she is healthy	21	12.1%
I am convinced that vaccinations are useless	18	10.3%
It is not recommended by doctors	16	9.2%
Others	9	5.2%
Unfriendly behavior of immunization staff	2	1.1%
If you are planning to vaccinate your daughter, what are your reasons?		
Vaccine endorsement by a physician	233	61.8%
Convinced that vaccine is effective	184	48.8%
Convinced that the vaccine is safe	179	47.5%
Others	9	2.4%
Trust in MOH	4	1.1%

Table 5 presents the factors associated with parental awareness regarding the HPV vaccine. Notably, 53.6% of parents with postgraduate degrees exhibited a good awareness of the vaccine, contrasting with 24% of individuals with lower educational levels (P = 0.049). Moreover, a significant difference in awareness was observed, with 75.4% of parents whose daughters' mothers received the HPV vaccine demonstrating good awareness, compared to only 32.6% among those whose daughters' mothers did not (P = 0.001). Additionally, good awareness regarding the vaccine was reported by 43.7% of parents whose daughters completed their immunizations according to the Ministry of Health's recommended schedule, in contrast to 26.6% of others (P = 0.009). Furthermore, all parents obtaining information from mass media displayed good awareness, while 90% of those relying on alternative sources, 80.6% consulting healthcare staff, and none without a specific source exhibited such awareness (P = 0.001).

**Table 5:** Factors associated with parents' awareness regarding HPV vaccine (n = 551). P: Pearson X2 test; $: Exact probability test; *: P < 0.05 (significant)

Overall awareness level						
Factors		Poor		Good		P value
		No	%	No	%	
Respondent	Mother	259	57.0%	195	43.0%	0.213
	Father	62	63.9%	35	36.1%	
	20-30	56	58.3%	40	41.7%	
Age of respondent	31-40	98	55.4%	79	44.6%	0.800
	41-50	119	59.8%	80	40.2%	
	> 50	48	60.8%	31	39.2%	
Nationality	Saudi	310	58.8%	217	41.2%	0.207
	Non-Saudi	11	45.8%	13	54.2%	
	Northern	47	55.3%	38	44.7%	
	Central	50	55.6%	40	44.4%	
Region	Eastern	51	62.2%	31	37.8%	0.883
	Western	133	59.1%	92	40.9%	
	Northern	40	58.0%	29	42.0%	
	Below secondary	19	76.0%	6	24.0%	
Educational level	Secondary	57	64.0%	32	36.0%	0.049*
	University	219	57.5%	162	42.5%	
	Postgraduate	26	46.4%	30	53.6%	
	Not working	136	60.2%	90	39.8%	
Work	Student	7	70.0%	3	30.0%	0.521^$^
	Working	178	56.5%	137	43.5%	
Marital status	Married	290	57.8%	212	42.2%	0.456
	Divorced/widow	31	63.3%	18	36.7%	
Did the mother (mother of the daughter) get the HPV vaccine?	Yes	29	24.6%	89	75.4%	0.001*
	No	292	67.4%	141	32.6%	
	Yes	21	21.9%	75	78.1%	0.155
Have you completed the HPV vaccination?	No	8	36.4%	14	63.6%	
Has your daughter received the HPV vaccine before?	Yes	38	26.4%	106	73.6%	0.001*
	No	283	69.5%	124	30.5%	
Has your daughter completed her immunizations according to the children's immunization schedule recommended by the Ministry of Health?	Yes	274	56.3%	213	43.7%	
	No	47	73.4%	17	26.6%	0.009*
	None	212	100.0%	0	0.0%	
	Healthcare staff	14	19.4%	58	80.6%	
	Social media	61	36.5%	106	63.5%	
	Internet websites	17	24.3%	53	75.7%	
Source of information	Mass media	0	0.0%	1	100.0%	0.001*^$^
	Health education campaigns	25	28.1%	64	71.9%	
	Other people	26	34.7%	49	65.3%	
	Daughter school	3	37.5%	5	62.5%	
	Others	1	10.0%	9	90.0%	

## Discussion

HPV is strongly implicated as a cause of cervical cancer, particularly during sexually active periods of life [6]. Cervical cancer ranks as the fourth most common cancer among women globally [12]. However, the majority of cancer-causing HPV infections can be prevented through vaccination and regular screening programs. The FDA has approved three HPV vaccines targeting carcinogenic HPV subtypes: the bivalent vaccine Cervarix, which targets subtypes 16 and 18; Gardasil, which expands coverage to include subtypes 6 and 11; and the recently authorized nine-valent HPV vaccine for females aged 9-26 and males aged 9-15. This nine-valent vaccine addresses five additional oncogenic HPV subtypes (31, 33, 45, 52, and 58), responsible for an additional 15% of cervical cancer cases [10].

Our research aimed to evaluate parental knowledge and attitudes toward the HPV vaccine. Additionally, we explored cultural influences contributing to vaccine hesitancy among parents. However, early education on HPV infection and associated risk factors positively impacted vaccination intentions [10].

This study revealed that over half of the parents knew of a vaccine against HPV. This finding contrasts significantly with a previous study by Alkalash et al., which reported only 32.9% awareness of the HPV vaccine among residents of the Western region of Saudi Arabia [13]. 

Approximately half of the study participants were aware that HPV has been established as the primary cause of most cervical cancers. However, another Saudi study [14] reported a higher prevalence of awareness (65%) regarding HPV's role in causing cervical cancer. However, a study done in India found poor knowledge and awareness levels regarding HPV and its vaccine [15]. Studies in Europe and sub-Saharan Africa revealed the same low level of awareness [16,17].

Similarly, half of the parents in our study knew that cervical cancer could be prevented with a vaccine. This finding is consistent with a previous study conducted in the Al-Ahsa region, where 67% of participants were unaware of the existence of HPV vaccination [18].

In this study, a significant majority (66.8%) of respondents knew that the HPV vaccine is recommended for females aged 9-26. This heightened awareness may be attributed to the early accessibility of HPV vaccines (bivalent and quadrivalent) in Saudi Arabia since 2010. These vaccines were integrated into the regular immunization schedules outlined in the Saudi National Immunization Schedule [19]. However, less than half of the respondents were familiar with the Ministry of Health's campaign promoting the HPV vaccine starting at age nine. This could be attributed to parents' focus primarily on the standard compulsory vaccinations for their children rather than newer and less conventional vaccines such as HPV. This underscores the importance of enhancing health education efforts among primary healthcare providers. Consequently, it is recommended to increase public awareness about the HPV vaccine by empowering healthcare providers in primary care settings [13].

In terms of overall parental knowledge, approximately half of the participants demonstrated a good awareness level regarding the HPV vaccine, while 58.3% exhibited a poor awareness level. This contrasts with a previous Saudi study, which reported a knowledge level of only 35% [14]. Moreover, higher levels of education were associated with better knowledge about HPV and its vaccine, a trend consistent with findings from other studies [9,20]. This association may be attributed to a greater willingness among individuals with higher education levels to accept new vaccines. Additionally, a notable proportion of mothers who had received the HPV vaccine exhibited good awareness. This heightened awareness significantly correlated with a stronger intent to vaccinate their daughters.

This study identified social media as the most frequently cited source of information regarding the HPV vaccine, consistent with findings from a previous study conducted in the East Province of Saudi Arabia [14]. Conversely, a study conducted in the Western region of Saudi Arabia found that physicians were the primary source of knowledge for participants [13]. When comparing our study with others conducted in Saudi Arabia, several similarities and differences regarding vaccine acceptance and hesitancy emerge. Our study indicates a higher percentage of parents intending to vaccinate their daughters compared to previous studies [9,10,14,19,21,22]. Consistent with another study [21], common reasons for hesitancy include fear of vaccines and needles. Similarly, our survey highlights the primary reasons for vaccine acceptance, such as physician endorsement, belief in vaccine efficacy, and safety. This aligns with findings from a previous study [13] where knowledgeable parents showed a greater willingness to vaccinate their children. However, misconceptions and barriers to HPV vaccine uptake persist across the studies, such as the belief in being healthy [14] and the confidence in not being at risk [13]. As previously reported, religious reasons also serve as a barrier [19].

We acknowledge several limitations in our study that merit consideration. Data collection relied on an online questionnaire, which may have implications for validity, particularly if the responses were researched or subject to recall bias. Furthermore, it is a cross-sectional design that demonstrates association and not a causal relationship.

## Conclusions

This community-based survey revealed a low level of awareness regarding the HPV vaccine among the parents of females aged 9-26 in Saudi Arabia. Most parents expressed intentions to vaccinate their daughters if they had not already received the HPV vaccine. Among those who expressed hesitancy, the most commonly reported reason for reluctance was a lack of prior awareness about the vaccine. These findings indicate a complex interplay of factors, including awareness, knowledge, cultural beliefs, risk perceptions, and healthcare interactions, shaping attitudes toward the HPV vaccine. Addressing these multifaceted factors has the potential to enhance vaccine acceptance and uptake.

## References

[1] (2024). Centers for disease control and prevention. STD facts - Human papillomavirus (HPV). https://www.cdc.gov/hpv/parents/about-hpv.html.

[2] (2024). World Health Organization. Sexually transmitted infections (STIs). https://www.who.int/health-topics/sexually-transmitted-infections#tab=tab_1.

[3] Bray F, Ferlay J, Soerjomataram I, Siegel RL, Torre LA, Jemal A (2018). Global cancer statistics 2018: GLOBOCAN estimates of incidence and mortality worldwide for 36 cancers in 185 countries. CA Cancer J Clin.

[4] Sait KH, Anfinan NM, Sait HK, Basalamah HA (2024). Human papillomavirus prevalence and dynamics: insights from a 5-year population-based study in Jeddah, Kingdom of Saudi Arabia. Saudi Med J.

[5] Ferlay J, Soerjomataram I, Dikshit R (2015). Cancer incidence and mortality worldwide: sources, methods and major patterns in GLOBOCAN 2012. Int J Cancer.

[6] Alsbeih G (2014). HPV infection in cervical and other cancers in Saudi Arabia: implication for prevention and vaccination. Front Oncol.

[7] Davies C, Stoney T, Hutton H (2021). School-based HPV vaccination positively impacts parents' attitudes toward adolescent vaccination. Vaccine.

[8] Della Polla G, Pelullo CP, Napolitano F, Angelillo IF (2020). HPV vaccine hesitancy among parents in Italy: a cross-sectional study. Hum Vaccin Immunother.

[9] Hussain AN, Alkhenizan A, McWalter P, Qazi N, Alshmassi A, Farooqi S, Abdulkarim A (2016). Attitudes and perceptions towards HPV vaccination among young women in Saudi Arabia. J Family Community Med.

[10] Alhusayn KO, Alkhenizan A, Abdulkarim A, Sultana H, Alsulaiman T, Alendijani Y (2022). Attitude and hesitancy of human papillomavirus vaccine among Saudi parents. J Family Med Prim Care.

[11] (2022). Chronic disease - cervical cancer. https://www.moh.gov.sa/en/awarenessplateform/ChronicDisease/Pages/CervicalCancer.aspx.

[12] (2022). Cervical cancer. http://cervical-cancer.

[13] Alkalash SH, Alshamrani FA, Alhashmi Alamer EH, Alrabi GM, Almazariqi FA, Shaynawy HM (2022). Parents' knowledge of and attitude toward the human papillomavirus vaccine in the western region of Saudi Arabia. Cureus.

[14] Almaghlouth AK, Bohamad AH, Alabbad RY, Alghanim JH, Alqattan DJ, Alkhalaf RA (2022). Acceptance, awareness, and knowledge of human papillomavirus vaccine in Eastern Province, Saudi Arabia. Cureus.

[15] Rashid S, Labani S, Das BC (2016). Knowledge, awareness and attitude on HPV, HPV vaccine and cervical cancer among the college students in India. PLoS One.

[16] López N, Garcés-Sánchez M, Panizo MB, de la Cueva IS, Artés MT, Ramos B, Cotarelo M (20201441). HPV knowledge and vaccine acceptance among European adolescents and their parents: a systematic literature review. Public Health Rev.

[17] Perlman S, Wamai RG, Bain PA, Welty T, Welty E, Ogembo JG (2014). Knowledge and awareness of HPV vaccine and acceptability to vaccinate in sub-Saharan Africa: a systematic review. PLoS One.

[18] Al-Darwish AA, Al-Naim AF, Al-Mulhim KS, Al-Otaibi NK, Morsi MS, Aleem AM (2014). Knowledge about cervical cancer early warning signs and symptoms, risk factors and vaccination among students at a medical school in Al-Ahsa, Kingdom of Saudi Arabia. Asian Pac J Cancer Prev.

[19] Darraj AI, Arishy AM, Alshamakhi AH (2022). Human papillomavirus knowledge and vaccine acceptability in Jazan Province, Saudi Arabia. Vaccines (Basel).

[20] Al Ghamdi NH (2022). Knowledge of human papilloma virus (HPV), HPV-vaccine and pap smear among adult Saudi women. J Family Med Prim Care.

[21] Gari A, Ghazzawi MA, Ghazzawi SA (2023). Knowledge about cervical cancer risk factors and human papilloma virus vaccine among Saudi women of childbearing age: a community-based cross-sectional study from Saudi Arabia. Vaccine X.

[22] Rezq KA, Algamdi M, Alanazi R, Alanazi S, Alhujairy F, Albalawi R, Al-Zamaa W (2023). Knowledge, perception, and acceptance of HPV vaccination and screening for cervical cancer among Saudi females: a cross-sectional study. Vaccines (Basel).

